# Pharmacodynamics, Mechanisms of Action and Resistance, and Spectrum of Activity of New Antifungal Agents

**DOI:** 10.3390/jof8080857

**Published:** 2022-08-16

**Authors:** Nathan P. Wiederhold

**Affiliations:** Fungus Testing Laboratory, Department of Pathology and Laboratory Medicine, University of Texas Health Science Center at San Antonio, San Antonio, TX 78229, USA; wiederholdn@uthscsa.edu

**Keywords:** pharmacodynamics, antifungals, mechanism of action, resistance, olorofim, manogepix, AT-2307, GR-2397, ibrexafungerp, rezafungin, oteseconazole, opelconazole, encochleate amphotericin B, MAT2203

## Abstract

Several new antifungals are currently in late-stage development, including those with novel pharmacodynamics/mechanisms of action that represent new antifungal classes (manogepix, olorofim, ATI-2307, GR-2397). Others include new agents within established classes or with mechanisms of action similar to clinically available antifungals (ibrexafungerp, rezafungin, oteseconazole, opelconazole, MAT2203) that have been modified in order to improve certain characteristics, including enhanced pharmacokinetics and greater specificity for fungal targets. Many of the antifungals under development also have activity against *Candida* and *Aspergillus* strains that have reduced susceptibility or acquired resistance to azoles and echinocandins, whereas others demonstrate activity against species that are intrinsically resistant to most clinically available antifungals. The tolerability and drug–drug interaction profiles of these new agents also appear to be promising, although the number of human subjects that have been exposed to many of these agents remains relatively small. Overall, these agents have the potential for expanding our antifungal armamentarium and improving clinical outcomes in patients with invasive mycoses.

## 1. Introduction

For decades, clinically available antifungals used to treat invasive mycoses have primarily targeted ergosterol, either by binding to it (i.e., polyenes) or through the inhibition of its biosynthesis (i.e., azoles). During the last 20 years, new members of the azoles, including the extended spectrum triazoles, voriconazole, posaconazole, and isavuconazole; lipid formulations of amphotericin B; and the echinocandins, which lack major toxicities and drug–drug interactions due to their fungal-specific mechanism of action, have become available and have led to improvements in clinical outcomes against certain mycoses [1,2]. However, these antifungals are not without limitations, including adverse effects/toxicities associated with amphotericin B and the azoles, and significant drug–drug interactions with the azoles due to interactions with mammalian cytochrome P450 (CYP450) enzymes [1]. The spectrum of activity of the echinocandins is narrow compared to amphotericin B and the azoles, and these antifungals must be administered intravenously, limiting their long-term use. The development of resistance is also of growing concern for the azoles and the echinocandins [3].

**Figure 1:**
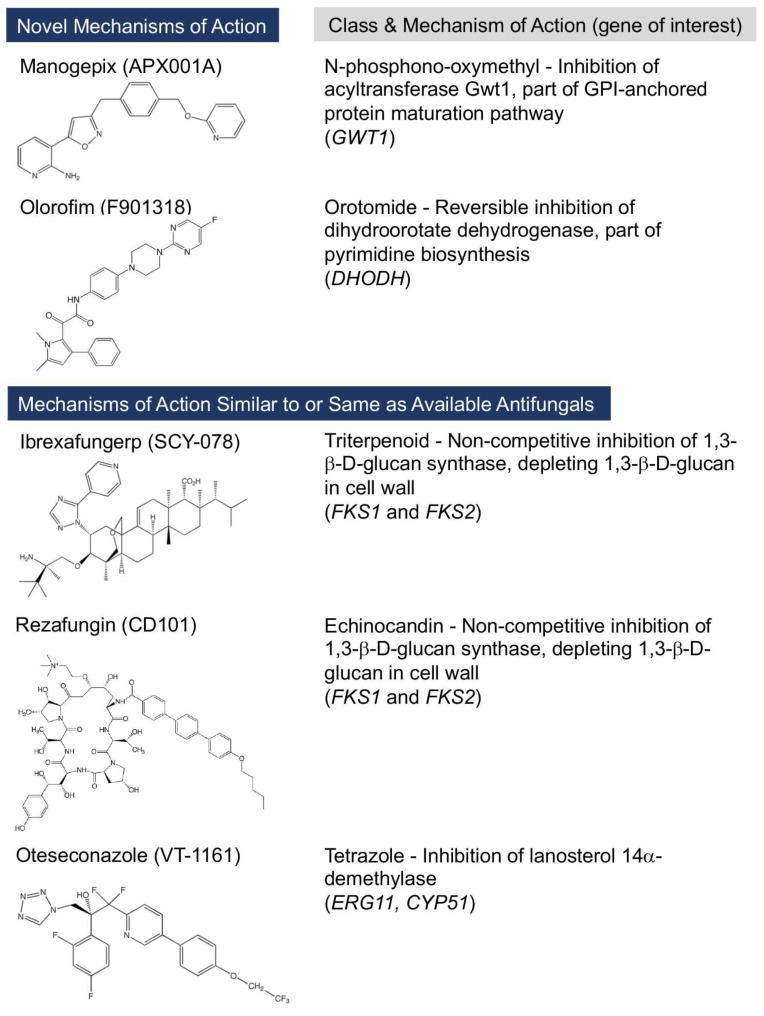
Chemical structures, antifungal classes, and mechanisms of action of antifungals currently under late-stage clinical development, including manogepix, olorofim, ibrexafungerp, rezafungin, and oteseconazole.

Currently, new antifungals with novel mechanisms of action are being developed, including those in Phase II and III clinical trials. These include manogepix, olorofim, ATI-2307, and GR-2397 [4,5]. In addition, other agents with mechanisms of action identical or similar to clinically available antifungals, but with distinct advantages, are also in development, including ibrexafungerp, rezafungin, oteseconazole, opelconazole, and the encochleate amphotericin B formulation, MAT2203. This review discusses the pharmacodynamics (i.e., mechanisms of action), mechanisms of resistance, spectrum of activity, and the pharmacokinetic/pharmacodynamic (PK/PD) parameters of these agents. In addition, their tolerability and drug–drug interaction profiles, as they relate to the mechanisms of action, are also reviewed. Those that are in late-stage clinical development or have recently been approved by the U.S. Food and Drug Administration for limited indications are shown in Table 1, and their structures/mechanisms of action and spectrums of activity are shown in Figure 1 and Table 2, respectively. The Clinical and Laboratory Standards Institute (CLSI) has also established and published acceptable minimum inhibitory concentration/minimum effective concentration (MIC/MEC) ranges and modal values for several of these agents (i.e., ibrexafungerp, manogepix, olorofim, and rezafungin) against quality control and reference strains that are available from the American Type Culture Collection (ATCC) [6,7].

## 2. Antifungals with Novel Mechanisms of Action

### 2.1. Manogepix

#### 2.1.1. Mechanism of Action—Pharmacodynamics

Manogepix (APX001A) acts against fungi by inhibiting the fungal acyltransferase enzyme, Gwt1, which is an important component of the glycosylphosphatidylinositol (GPI)-anchored protein maturation pathway, and is essential for trafficking mannoproteins to the fungal cell membrane and wall [8,9]. GPI-anchored mannoproteins serve as adhesions, enabling fungi to adhere to mucosal epithelial surfaces within the host prior to colonization, as well as infection [10]. Some fungal virulence factors are also derived from GPI-anchored proteins [10,11,12,13,14]. Thus, the inhibition of their synthesis may have pleotropic effects aside from growth inhibition. This agent was identified through a targeted search for agents that specifically inhibit Gwt1 and the optimization of identified leads [15]. Clinically, manogepix is administered as the N-phosphonooxymethyl prodrug, fosmanogepix (APX001), which is rapidly converted to manogepix by host phosphatases [15,16,17].

#### 2.1.2. Spectrum of Activity and Resistance

Manogepix has broad-spectrum in vitro activity against fungi. Against yeasts, this includes activity against most *Candida* species, including *C. albicans, C. auris, C. glabrata, C. parapsilosis,* and *C. tropicalis*, as well as azole- and echinocandin-resistant strains [16,17,18,19,20], and *Cryptococcus neoformans* and *C. gattii* [21]. This in vitro activity has translated into efficacy in experimental models of candidiasis and cryptococcosis, including against infections caused by strains resistant to clinically available antifungals [20,21,22,23]. However, manogepix lacks or has limited activity against *Candida krusei, C. inconspicua,* and *C. kefyr* (*Kluyveromyces marxianus*). Activity has also been demonstrated against a limited number of *Rhodotorula* isolates, whereas its activity against *Trichosporon asahii* was variable [17].

Manogepix is also active against pathogenic molds, including *Aspergillus, Fusarium,* and *Scedosporium* species, as well as *Lomentospora* (*Scedosporium*) *prolificans,* a pathogen that is intrinsically resistant to clinically available antifungals [8,24,25,26,27,28,29]. Potent activity is also observed against strains of azole-resistant *A. fumigatus*. Similar to the echinocandins, manogepix does not necessarily inhibit the growth of filamentous fungi, but rather causes morphologic changes, which are observed in vitro as short, stubby, abnormally-branched hyphae, and the lowest concentration at which these occur is referred to as the MEC [15,30]. Reports of activity against different members of the order Mucorales have been mixed, with some studies showing no in vitro activity, but others reporting limited in vitro and in vivo activity against *Rhizopus arrhizus* [8,19,31]. A recent study reported that combination therapy with liposomal amphotericin B and fosmanogepix was superior to either agent alone against invasive aspergillosis, fusariosis, and mucormycosis [32].

Resistance to manogepix can develop due to point mutations that lead to amino acid substitutions within Gwt1 (i.e., V163A in *C. glabrata* and V162A in *C. albicans*) [33], and these changes do not affect the activity of other antifungals, such as the azoles and echinocandins. Interestingly, strains with elevated manogepix MICs that are wild-type for Gwt1 have also been reported to be cross-resistant to fluconazole. Although such cross-resistance has been attributed to efflux pumps due to marked increases in the transcription of efflux pump genes, such as *CDR11, SNQ2*, and *MDR1* in *C. albicans* and *MDR1* in *C. parapsilosis* [34], changes in manogepix and fluconazole MICs were minimal.

#### 2.1.3. In Vivo Efficacy and Pharmacokinetics/Pharmacodynamics

The in vitro activity of manogepix has translated into in vivo efficacy in animal models of aspergillosis, fusariosis, scedoporiosis, coccidioidomycosis, and mucormycosis caused by *Rhizopus arrhizus* [24,27,31,35,36]. Interestingly, a recent study reported that combination therapy with liposomal amphotericin B and fosmanogepix was superior to either agent alone against invasive aspergillosis, fusariosis, and mucormycosis [32].

In experimental models of invasive candidiasis caused by *C. albicans, C. glabrata,* and *C. auris*, as well as invasive aspergillosis due to both wild-type and azole-resistant *A. fumigatus,* the pharmacokinetic/pharmacodynamic (PK/PD) parameters associated with manogepix efficacy were the AUC/MIC and AUC/MEC, respectively [20,23,35,37]. Against invasive candidiasis, the total free drug AUC/MIC (fAUC/MIC) ratios associated with stasis ranged from 1.35 to 22.54 [23]. Similarly, against invasive aspergillosis, the median fAUC/MEC associated with a 1-log reduction in fungal burden was 89.39 [35].

#### 2.1.4. Tolerability and Drug Interactions

Manogepix appears to have fungal-specific activity, as it does not inhibit the human inositol acyltransferase, Pigw, at clinically relevant concentrations [38]. Administration of fosmanogepix has been safe and well-tolerated in Phase I and II clinicals trials, with only mild adverse effects reported [39,40,41,42]. Clinically significant adverse effects and dose-limiting toxicities have not been observed. A Phase I drug–drug interaction study evaluating the effects of CYP3A4 and pan-CYP450 inhibition on fosmanogepix has been completed, but the results are not yet available [15]. However, the inhibition of CYP3A4 may potentially be of concern, as the non-selective CYP450 inhibitor, 1-aminobenzotriazole, has been utilized to improve the overall exposure profile of manogepix in mice due to rapid metabolism of this agent in this animal species [24,37,43].

### 2.2. Olorofim

#### 2.2.1. Mechanism of Action—Pharmacodynamics

Olorofim (F901318, orotomide class) reversibly inhibits the dihydroorotate dehydrogenase (DHODH) enzyme, which is involved in the biosynthesis of pyrimidine [44]. This disruption leads to the loss of uridine-5′-monophosphate (UMP) and uridine-5′-triphosphate (UTP), which are important for the production of various cell wall components, as well as cytosine, thymine, and uracil, and also in cell cycle regulation [45]. Exposure of fungi to olorofim results in cell cycle arrest, as well as cell lysis [46,47]. Other effects have also been observed in *Aspergillus* following exposure to this agent, including inhibition of conidial germination; slowing of germ tube and hyphal growth; hyphal lysis; and, finally, cell death with prolonged exposure (120 h), suggesting that the effects of olorofim change from fungistatic to fungicidal with longer exposures [46]. Other effects of olorofim exposure include increases in hyphal septation, reductions in hyphal compartment sizes, and increased vacuolar volume, with the latter possibly indicating cell cycle arrest and a sign of autophagy [47,48]. This agent was identified through a screen of a library containing over 340,000 small molecules for in vitro activity specifically against *Aspergillus fumigatus* [44].

#### 2.2.2. Spectrum of Activity and Mechanisms of Resistance

The spectrum of activity of olorofim is unique. It demonstrates potent activity against several pathogenic molds and dimorphic fungi, including *Blastomyces, Coccidioides,* and *Histoplasma* species, but it is devoid of activity against the Mucorales and yeasts, including *Candida* and *Cryptococcus* species [44,49,50,51,52,53]. In addition, the activity of olorofim is not uniform against all molds. For example, species-specific activity has been observed against *Fusarium* species, with the least activity observed against members of the *Fusarium solani* species complex [44,52]. This unique spectrum of activity of olorofim has been attributed to differences in the DHODH enzymes among various groups of fungi [44].

Despite the lack of activity against several important pathogen groups, olorofim has demonstrated promising activity against several fungi that either have reduced susceptibility or resistance to the extended spectrum azoles and amphotericin B. This includes azole-resistant *Aspergillus fumigatus* strains with *CYP51A* gene mutations, and cryptic *Aspergillus* species (e.g., *A. calidoustus*, *A. lentulus*, *A. tanneri*, *A. thermomutatus*, and *A. udagawae*) with reduced azole susceptibility [44,53,54,55]. Olorofim is also active against several other molds that have reduced susceptibility or resistance to azoles and amphotericin B, including *Scedosporium* species, *Lomentospora prolificans,* and *Microascus/Scopulariopsis* species [44,49,51,52]. Other fungi that are inhibited by this agent include the hyaline molds, *Paecilomyces variotii* and *Talaromyces marneffei,* among others, and *Madurella mycetomatis*, the most common cause of eumycotic mycetoma [44,50,56].

Olorofim resistance can develop secondary to mutations within the gene encoding for DHODH. In a screen of 975 *A. fumigatus* isolates, no intrinsic resistance to this agent was found [57]. However, isolates with olorofim MICs of >8 mg/L could be selected in the laboratory with higher inocula and longer exposures to this agent. This was attributed to mutations within the *PYRE* gene leading to amino acid substitutions at locus G119 (i.e., G119C and G119V) within DHODH, and a reduced affinity of olorofim for the mutated protein. Although fungi are capable of scavenging pyrimidine from the environment, the concentrations needed to reverse the in vitro effects of olorofim (≥5 mM) are markedly higher than what is found in human serum (~15 μM) [44]. Thus, the use of exogenous pyrimidine does not appear to be a mechanism by which fungi may become resistant to olorofim.

#### 2.2.3. In Vivo Efficacy and Pharmacokinetics/Pharmacodynamics

Olorofim has also demonstrated in vivo effectiveness in several experimental models of invasive fungal infections, including invasive aspergillosis caused by different species of *Aspergillus* and azole-resistant *A. fumigatus* strains, central nervous system coccidioidomycosis, and disseminated scedosporiosis and lomentosporiosis [50,54,58,59,60]. The PK/PD parameter of olorofim associated with efficacy in experimental models of invasive mycoses has been the trough or Cmin/MIC. Against invasive aspergillosis caused by either azole-susceptible or resistant *A. fumigatus* strains, reductions in serum galactomannan levels and improvements in survival occurred with more frequent administration, and this time-dependent activity was confirmed by dose-fractionation studies [58]. A reduction in galactomannan of 27% was achieved with a Cmin/MIC range of 3 to 16.5 against 8 *A. fumigatus* challenge strains. These findings have been confirmed in other models of invasive mycoses, including sinopulmonary aspergillosis due to *A. flavus*, and coccidioidal meningitis caused by *C. immitis* [50,59], and is consistent with the time-dependent activity described in vitro [46].

#### 2.2.4. Tolerability and Drug Interactions

Olorofim is significantly more potent against *A. fumigatus* DHODH (IC50 44 nM) compared to recombinant human DHODH (IC50 > 100 μM) [44], suggesting fungal-specific activity. In Phase I clinical studies, no significant changes in vital signs or laboratory values were reported, and no severe or serious adverse effects were observed in healthy subjects administered this agent [61,62]. Olorofim does undergo Phase I hepatic metabolism by several CYP450 enzymes [63], and it also acts as a weak inhibitor of CYP3A4 [64]. Thus, drug–drug interactions may be a clinical concern with this agent.

### 2.3. Other Antifungals with Novel Mechanisms of Action

Other antifungals with novel mechanisms of action that are not yet at advanced stages of clinical development include ATI-2307 and GR-2307. ATI-2307 (formerly T-2307) is an aromatic diamidine with a structure similar to pentamidine that was identified in a screen of specific compounds in the chemical library at Toyama Chemical Company [65]. This agent causes the collapse of fungal mitochondrial membrane potential by inhibiting the respiratory chain complex, resulting in decreased adenosine triphosphate levels [66,67,68]. ATI-2307 has in vitro activity against *Candida* species, including azole and echinocandin-resistant strains of *C. albicans, C. glabrata,* and *C. auris, Cryptococcus* species, *Malassezia furfur, Aspergillus* species, *Fusarium solani*, and *Lichtheimia* *corymbifera* [65,69,70,71,72]. In vivo efficacy has also been reported in animal models of invasive candidiasis, cryptococcosis, and aspergillosis [65,70,71,73,74]. However, reduced to no activity has been reported against *Rhizopus arrhizus, Mucor racemosus, Scedosporium* species, *Trichophyton rubrum,* and *Trichosporon asahii.*

GR-2397, formerly VL-2397 and ASP2397, is a cyclic hexapeptide originally isolated from an *Acremonium persicinum* strain (MF-34833) as part of a program to discover new agents for pulmonary aspergillosis [75]. Although the intracellular drug target is unknown, GR-2397 is structurally related to the siderophore, ferrichrome, and this agent is taken up into *A. fumigatus* cells by the siderophore transporter, Sit1 [76]. Thus, the antifungal activity is observed against species in which Sit1 is present [63,77], such as *C. glabrata*, including echinocandin- and azole-resistant strains, and *C. kefyr,* but not *C. albicans* [76,77,78]. Activity has also been demonstrated against *Aspergillus* species, including azole-susceptible and resistant *A. fumigatus* strains, *A. flavus,* and *A. terreus* [76,79]. In experimental models of invasive candidiasis, efficacy has been demonstrated against infections caused by both wild-type and azole- and echinocandin-resistant *C. glabrata* isolates [78]. Efficacy has also been reported against aspergillosis [76], and the limited PK/PD data that are available suggest that AUC/MIC is the parameter most closely associated with in vivo efficacy, as this was demonstrated in a murine model of invasive pulmonary aspergillosis [80].

## 3. New Antifungals That Improve upon Current Classes and Mechanisms of Action

### 3.1. Ibrexafungerp

#### 3.1.1. Mechanism of Action—Pharmacodynamics

Ibrexafungerp (SCY-078, triterpenoid class) is a semi-synthetic compound derived from the natural product, enfumafungin [81]. Similar to the echinocandins, ibrexafungerp inhibits the production of 1,3-β-d-glucan through non-competitive inhibition of the 1,3-β-d-glucan synthase complex [82,83], although it is structurally different and not a member of this class. Inhibition of 1,3-β-d-glucan synthesis weakens the cell wall, and results in osmotic instability and eventual cell lysis [82,84]. However, the binding sites for ibrexafungerp and the echinocandins only partially overlap; thus, cross-resistance between these different antifungal classes is limited [85,86,87]. In addition, unlike the echinocandins, ibrexafungerp can be absorbed from the gastrointestinal tract following oral administration, and does not have to be administered intravenously.

#### 3.1.2. Spectrum of Activity and Mechanisms of Resistance

Ibrexafungerp has in vitro activity against several *Candida* species, including *C. albicans, C. glabrata*, *C. parapsilosis*, and *C. tropicalis,* as well as the emerging pathogen, *C. auris* [88]; against which, antibiofilm activity has also been demonstrated [89,90]. Its activity is also maintained against azole-resistant isolates. However, reduced potency has been reported against *C. lusitaniae* and *C. krusei*. Unlike the azoles, the activity of ibrexafungerp against *Candida* species is maintained in low pH environments [91,92,93], which may make it useful for the treatment of vulvovaginal candidiasis, for which it has received regulatory approval for clinical use in the U.S.

Ibrexafungerp also demonstrates activity against *Aspergillus* species, including *A. fumigatus*, *A. niger*, and *A. terreus*, as well as cryptic species and strains that are azole-resistant [83,85,94,95]. In a rabbit model of invasive aspergillosis, enhanced efficacy, as measured by reductions in pulmonary injury, fungal burden, galactomannan and 1,3-β-d-glucan levels, and improvements in survival, was observed when ibrexafungerp was combined with isavuconazole [96]. However, ibrexafungerp lacks in vitro activity against the Mucorales and *Fusarium* species, and has variable activity against other molds, including *Microascus/Scopulariopsis* species, *Purpureocillium lilacinum,* and *Scedosporium* species [97]. The in vitro activity of ibrexafungerp and the echinocandins against molds is measured as the MEC value, which, as described above for manogepix, is the lowest concentration that results in morphologic changes (i.e., short, stubby, abnormally-branched hyphae) [30]. These changes are due to the location of the 1,3-β-d-glucan synthase enzymes at the apical tips and branch points of hyphae where growth occurs [30,82].

Point mutations within the *FKS1* and *FKS2* genes that encode subunits of 1,3-β-d-glucan synthase can lead to ibrexafungerp and echinocandin resistance [98]. As noted previously, the binding sites for ibrexafungerp and the echinocandins only partially overlap. Thus, the activity of ibrexafungerp against *Candida* strains harboring *FKS* mutations is variable [87,99], although it is generally more potent against *FKS* mutants compared to the echinocandins [99,100,101,102]. However, *FKS* mutations that lead to specific amino acid changes, such as F641S in *C. albicans*, and F649del, F658del, F659S, F659del, E655A, and W715L in *C. glabrata*, can reduce ibrexafungerp activity [95,103,104,105,106].

#### 3.1.3. In Vivo Efficacy and Pharmacokinetics/Pharmacodynamics

Consistent with its in vitro activity, several studies have reported ibrexafungerp to have in vivo efficacy in experimental models in candidiasis caused by different *Candida* species, including *C. albicans, C. glabrata, C. tropicalis*, and *C. auris* [107,108]. Ibrexafungerp has also demonstrated prophylactic activity in a murine model against *Pneumocystis* pneumonia due to its activity against the cyst form of this organism [109]. From a PK/PD standpoint, the AUC/MIC has correlated with the effectiveness of ibrexafungerp in animal models of invasive candidiasis [107,110]. When assessed by overall exposure, the ibrexafungerp free drug AUC/MIC (fAUC/MIC) ratios associated with stasis, and measured by reductions in kidney fungal burden, have ranged from 0.1 to 1.7, and these were lower to those reported for the echinocandins [107,110,111]. This difference may be due to higher concentrations of ibrexafungerp within the kidneys due to extensive tissue distribution [110]. Fungicidal activity, defined as a 1-log_10_ reduction in fungal burden, has been reported with ibrexafungerp fAUC/MIC ratios of 0.91 to 1.42 [107]. The estimated protein-binding of ibrexafungerp ranges between 99.5% and 99.8% [110]. Although the PK/PD parameter of ibrexafungerp has not been formally defined against *Aspergillus* infections, it is thought to be the AUC/MEC [112].

#### 3.1.4. Tolerability and Drug Interactions

In Phase I and II clinical studies, ibrexafungerp has been well-tolerated, although non-serious adverse effects did increase with higher doses and longer durations of therapy [113]. The most common mild-to-moderate adverse effects included nausea, vomiting, diarrhea, and abdominal pain [112,114,115]. Of note, prolongations in QTc intervals were not observed in a Phase II study of patients with invasive candidiasis [115]. Ibrexafungerp is metabolized by CYP3A4, and the coadministration of strong inducers, such as rifampin, or inhibitors of this enzyme, including itraconazole, should be avoided, as these may lead to insufficient or supratherapeutic ibrexafungerp concentrations, respectively. Ibrexafungerp is also a reversible inhibitor of CYP2C8 and 3A4. However, the coadministration of ibrexafungerp and rosiglitazone, which is metabolized by CYP2C8, had no effect on the overall exposure of this anti-diabetic drug [116]. Similarly, only a modest increase in tacrolimus concentrations, which is metabolized by CYP3A4, were observed with the co-administration of ibrexafungerp [112]. Although tacrolimus dose adjustments are not currently recommended when co-administered with ibrexafungerp, levels of this calcineurin inhibitor should be monitored.

### 3.2. Rezafungin

#### 3.2.1. Mechanism of Action—Pharmacodynamics

Rezafungin (CD101) is a second-generation echinocandin, and, similar to other echinocandins, causes non-competitive inhibition of the 1,3-β-d-glucan synthase enzyme complex [117]. 1,3-β-d-glucan is a major cell wall component of many pathogenic fungi; thus, inhibiting its synthesis results in osmotic instability and eventual cell lysis [82,84]. Rezafungin is similar to anidulafungin in structure, but is modified within the cyclic core, having the ornithine hemiaminal replaced with a choline aminal ether [118]. This change leads to greater stability and a prolonged half-life for rezafungin (~130 h vs. ~24 h for anidulafungin) [118,119,120,121,122].

#### 3.2.2. Spectrum of Activity and Mechanisms of Resistance

As with the other echinocandins, rezafungin has potent in vitro activity against *Candida* and *Aspergillus* species. This includes *Candida* species that frequently cause infections in humans, such as *C. albicans, C. glabrata, C. tropicalis,* and *C. krusei*, as well as less common species, including *C. dubliniensis, C. fabianii, C. inconspicua, C. kefyr, C. lipolytica,* and *C. lusitaniae*, among others [123,124,125,126]. Potent activity has also been observed against *C. auris* [126]. Reduced potency is observed against members of the *C. parapsilosis* species complex, including *C. parapsilosis sensu stricto, C. orthopsilosis*, and *C. metapsilosis*, as well as against *C. guilliermondii* [125,126]. Rezafungin also lacks activity against fungi that are intrinsically resistant to the echinocandin class, including *Cryptococcus, Rhodotorula*, and *Trichosporon* species [117,124]. Rezafungin does have activity against *Aspergillus fumigatus,* including azole-resistant isolates, *A. flavus, A. terreus,* and *A. niger*. It is also active against cryptic members of *Aspergillus* section *Fumigati* (e.g., *A. lentulus, A. thermomutatus*, and *A. udagawae*), and *A. calidoustus* [123,127]. As with the other echinocandins, the in vitro activity of rezafungin against molds is measured as the MEC and not the MIC. Resistance to the echinocandins is caused by mutations within highly conserved regions (hot spots 1 and 2) of *FKS1* and *FKS2* genes that encode subunits of the 1,3-β-d-glucan synthase complex [98]. In fact, the reduced activity of this class against members of the *C. parapsilosis* species complex and *C. guilliermondii* is due to naturally occurring point mutations within *FKS1* [128,129]. Against *Candida* isolates harboring *FKS* mutations, rezafungin MICs have been reported to be similar to those of other echinocandins [124,130].

#### 3.2.3. In Vivo Efficacy and Pharmacokinetics/Pharmacodynamics

In vivo efficacy has also been reported against infections caused by *Candida* and *Aspergillus* species. Experimental models of invasive candidiasis have demonstrated rezafungin to be effective against infections caused by several *Candida* species, including *C. albicans, C. auris, C. dubliniensis, C. glabrata, C. parapsilosis,* and *C. tropicalis* [23,131,132,133,134,135]. Although its in vitro activity appears to be similarly affected by *FKS* mutations in a similar fashion as the other echinocandins, in one study, rezafungin maintained in vivo efficacy against infection caused by a *C. albicans* isolate harboring a heterozygous *FKS* mutant at codon S645, despite reduced in vitro activity against this strain [135]. Rezafungin was also effective in a rabbit model for the treatment of endophthalmitis caused by wild-type *C. albicans* [136]. In experimental models of invasive aspergillosis caused by *A. fumigatus,* rezafungin was also effective against infections caused by wild-type and azole-resistant strains [137,138]. Prophylactic efficacy has also been demonstrated against *Pneumocystis* pneumonia, as rezafungin was effective in blocking the formation of the reproductive forms of *P. murina* in immunocompromised mice [139].

As with the other echinocandins, the PK/PD parameter associated with rezafungin efficacy is the AUC/MIC. In neutropenic murine models of invasive candidiasis caused by *C. albicans, C. glabrata,* and *C. parapsilosis* strains with varying echinocandin susceptibility profiles, free drug AUC/MIC ratios associated with fungal burden stasis ranged from 0.07 to 2.92, whereas 1-log_10_ reductions in fungal burden were two- to four-fold higher [131]. Similarly, in murine models of candidiasis caused by *C. auris, C. dubliniensis,* and *C. tropicalis*, free drug AUC/MIC ratios associated with statis ranged from 1.88 to 11.65, and those associated with at least a 1-log_10_ reduction in fungal burden were also approximately two- to four-fold higher [131,132]. Dose fractionation studies in mice have also demonstrated that the shape of the concentration–time curve is important against *Candida* infections, as single high doses were associated with the largest decreases in fungal burden compared to the same overall doses administered more frequently [140]. The Cmax/MEC ratio has been reported to be associated with efficacy for the echinocandins against *A. fumigatus* infections [141], and extended-interval dosing of rezafungin was associated with improved survival and reductions in fungal burden in a murine model of disseminated aspergillosis caused by an *A. fumigatus* isolate harboring a TR_34_/L98H mutation in *CYP51A* [138].

#### 3.2.4. Tolerability and Drug Interactions

Due to the fungal-specific mechanism of action of the echinocandins, this class is very well-tolerated. In healthy volunteers, the majority of adverse effects were mild and transient. There was a tendency towards higher rates of adverse effects, such as infusion reactions, in the group that received the highest rezafungin dose [121]. In addition, no ECG abnormalities, including prolongations the QTc interval, were reported with rezafungin infusions up to 1400 mg [142]. Similarly, in Phase II studies, rezafungin was safe and well-tolerated in patients with candidemia and invasive candidiasis, and treatment-emergent adverse effects were deemed to be mild to moderate [143]. Rezafungin has a low potential for drug–drug interactions, and minimal interactions with recombinant CYP450 enzymes have been observed in vitro [144].

### 3.3. Oteseconazole

#### 3.3.1. Mechanism of Action—Pharmacodynamics

To overcome drug–drug interactions that limit the clinical utility of triazoles, oteseconazole (VT-1161) and similar compounds (e.g., VT-1129 and VT-1598) have been designed to have greater specificity for the fungal Cyp51 enzyme (i.e., lanosterol 14α-demethylase). In oteseconazole, the triazole iron-binding group has been replaced with a tetrazole (i.e., four nitrogen atoms in the five-member ring), and the portion of the molecule recognized by amino acids of the substrate-binding site within Cyp51 has also been modified [145]. Studies have reported a greater affinity of oteseconazole for fungal Cyp51 compared to human CYP450 enzymes (~2000-fold) [145,146,147,148]. Thus, oteseconazole’s mechanism of action is the same as the triazoles (i.e., inhibition of ergosterol biosynthesis), but with greater selectivity for fungal enzymes and potentially fewer adverse effects and drug–drug interactions.

#### 3.3.2. Spectrum of Activity and Mechanisms of Resistance

Oteseconazole is active against *Candida* species, including fluconazole-susceptible and resistant isolates, *Cryptococcus neoformans*, *Coccidioides immitis/posadasii*, and *Trichophyton* species [148,149,150,151,152,153].

Resistance to oteseconazole can occur by some of the same mechanisms that cause resistance to the triazoles. In *Candida albicans*, marked increases in oteseconazole MICs were reported to be caused by different mechanisms, including a premature stop codon in the *ERG3* gene; amino acid substitutions within the Erg11 enzyme; and overexpression of the ATP-binding cassette transporter genes, *CDR1* and *MDR1* [152,154]. Similarly, in *C. glabrata*, the efflux pump, Cdr1p, appears to affect oteseconazole activity to a greater extent than that of Pdh1 and Snq2, all of which are regulated by the zinc cluster transcription factor, Pdr1 [151]. Oteseconazole activity was also affected by Upc2a, another zinc cluster transcription factor that regulates the genes involved in ergosterol biosynthesis.

#### 3.3.3. In Vivo Efficacy and Pharmacokinetics/Pharmacodynamics

Efficacy has also been reported in animal models of various mycoses, including oropharyngeal and vulvovaginal candidiasis caused by fluconazole-susceptible and -resistant *Candida albicans* strains, and onychomycosis [155,156,157]. Reductions in fungal burden and improvements in survival were also reported with oteseconazole treatment in experimental models of coccidioidomycosis [149,158]. Prophylactic efficacy has also been demonstrated in a murine model of pulmonary mucormycosis caused by *Rhizopus arrhizus* var. *arrhizus*, which is consistent with the in vitro activity demonstrated against this species [159].

Although the PK/PD profile of oteseconazole most closely associated with efficacy has not been formally studied, it is likely to be similar to the azoles, which is the AUC/MIC. Given that the half-life reported in humans is 138 days, good exposures are expected [160,161]. Long half-lives leading to sustained plasma levels and exposures have been observed in various animals [149,158,161], and in a guinea pig model of onychomycosis, efficacy was observed with either once-daily or once-weekly dosing [156]. Oteseconazole was recently approved by the U.S. Food and Drug Administration for the treatment of recurrent vulvovaginal candidiasis, and is recommended to be given orally at doses of 600 mg on day 1; 450 mg on day 2; and then, beginning on day 14, 150 mg once-weekly when used as monotherapy [160].

#### 3.3.4. Tolerability and Drug Interactions

Oteseconazole has been well-tolerated in clinical trials, with the most frequently reported adverse effects being headache and nausea [160,161,162,163,164]. Most adverse effects were mild to moderate, and were judged to be unrelated to the study drug. Oteseconazole does not undergo significant metabolism, and co-administration with other drugs that are metabolized by CYP3A4 (midazolam, ethinyl estradiol, norethindrone) or are substrates of p-glycoprotein (digoxin) did not result in significant differences in the pharmacokinetics of these agents [160]. These results are consistent with oteseconazole having greater selectivity for fungal Cyp51 compared to mammalian CYP450 enzymes.

## 4. New Routes of Administration

In addition to new classes of antifungals with novel mechanisms of action and modifications to established classes, new formulations of antifungals within established classes that have different routes of administration are also being developed, including opelconazole and encochleate amphotericin B (MAT2203). Opelconanzole (PC945) is a triazole under development for administration directly to the lungs via inhalation, thus possibly limiting systemic exposure [165]. Thus, it may reach high pulmonary concentrations at the site of infection of fungi while possibly avoiding drug–drug interactions and adverse effects that occur primarily within the liver. Opelconazole has broad-spectrum activity against *Candida* species, including *C. albicans, C. glabrata, C. krusei,* and *C. auris*; *Cryptococcus* species; several *Aspergillus* species, including *A. fumigatus* and *A. flavus*; and *Rhizopus arrhizus* [166]. However, it lacks activity against other fungal pathogens, including *A. niger, Lichtheimia corymbifera,* and certain *Penicillium* species (i.e., *P. chrysogenum* and *P. citrinum*). Studies in humans have been limited, but opelconazole has been well-tolerated following inhaled administration to healthy individuals and those with mild asthma [165].

MAT2203 is a nanoparticle-based, encochleated formulation of amphotericin B that is under development for oral administration of this polyene. As with other amphotericin B formulations, MAT2203 binds to ergosterol within the fungal cell membrane, leading to membrane disruption. Amphotericin B has broad-spectrum activity, and MAT2203 was reported to have dose-dependent activity in different organs in a murine model of systemic candidiasis [167]. In humans, measurable bloodstream concentrations have been reported following oral administration of MAT2203, and the agent was well-tolerated at doses up to 800 mg/day [168].

## 5. Conclusions

Currently, there are several antifungals in development for the treatment of invasive mycoses, with some in late-stage clinical trials. These include agents within new classes with novel mechanisms of action and pharmacodynamics (i.e., manogepix, olorofim, ATI-2307, and GR-2397) that demonstrate in vitro and in vivo activity against strains that have developed resistance to azoles and the echinocandins, as well as species that are intrinsically resistant to most clinically available antifungals. Others include those with the same or similar mechanisms of action of clinically available antifungals, but that have been modified to improve their pharmacokinetic profile (i.e., rezafungin), allow for oral administration (i.e., ibrexafungerp, MAT2203), are delivered directly to the lungs (opelconazole), or have reduced potential for clinically significant drug–drug interactions and adverse effects (i.e., oteseconazole). Each of these agents has the potential to improve clinical outcomes and expand options available to clinicians for the treatment of invasive mycoses.

## Figures and Tables

**Table 1:** Antifungals in late-stage clinical development, routes of administration, pharmacokinetic/pharmacodynamic (PK/PD) parameters associated with efficacy, tolerability/adverse effects and drug interactions, and current clinical trials. Cmax = peak bloodstream concentration; Cmin = trough bloodstream concentration; AUC = area under concentration curve; fAUC = free drug area under concentration curve; MIC = minimum inhibitory concentration; MEC = minimum effective concentration; CYP450 = cytochrome P450 enzyme; CYP3A4 = cytochrome P450 3A4 enzyme. Information regarding current clinical trial status was obtained from https://clinicaltrials.gov/ (accessed on 8 July 2022).

Agent and Company Developing	Routes of Administration	PK/PD Parameter Associated with In Vivo Efficacy	Tolerability/Adverse Effects and Drug Interactions	Current Clinical Trials (Number and Phase)
Manogepix (APX001A) Pfizer	Intravenous and oral	AUC/MIC vs. yeasts (fAUC/MIC 1.35–22.54)	Well-tolerated in Phase I and II clinical studies	Candidemia/invasive candidiasis (NCT05421858, Phase III)
		AUC/MEC vs. molds (fAUC/MEC 89.39)	Drug interaction profile not yet known	
Olorofim (F901318) F2G	Intravenous and oral	Cmin/MIC (Cmin/MIC 3–16.5 vs. *A. fumigatus*)	Well-tolerated in Phase I studies with no serious adverse effects	Invasive aspergillois (NCT05101187, Phase III)
			Potential for drug interactions, as it is metabolized by CYP450 enzymes and is a weak inhibitor of CYP3A4	Aspergillosis, lomentosporiosis, scedosporiosis, and other resistant fungi (NCT03583164, Phase II)
Ibrexafungerp (SCY-078) Scynexis	Oral (Intravenous formulation under development)	AUC/MIC vs. *Candida (fAUC/MIC 0.1–1.7)*	Well-tolerated in Phase I and II clinical studies, and no QTc prolongations reported	Complicated vulvovaginal candidiasis (NCT05399641, Phase III)
				Invasive pulmonary aspergillosis (NCT03672292, Phase III)
		AUC/MIC Possibly AUC/MEC vs. Aspergillus	Potential for drug interactions, as it is metabolized by CYP3A4 and is also an inhibitor of CYP2C8 and 3A4	Candida auris candidiasis (NCT03363841, Phase III)
				Invasive mycoses in those who are refractory to or intolerant of other therapies (NCT03059992, Phase III)
Rezafungin (CD101) Cidara	Intravenous	AUC/MIC vs. *Candida (fAUC/MIC 0.07–11.65)*	Well-tolerated in Phase I and II clinical studies; some infusion-related reactions with higher doses	Antifungal prophylaxis in adults undergoing allogeneic stem cell transplantation (NCT04368559, Phase III)
		AUC/MEC and Cmax/MEC vs. Aspergillus	Low potential for drug interactions	
Oteseconazole (VT-1161) Mycovia	Oral	Undefined, but most likely AUC/MIC (similar to triazoles)	Well-tolerated in Phase I and II clinical studies with mild-to-moderate adverse effects	Recurrent vulvovaginal candidiasis (NCT03562156 and NCT033561701, Phase III—Completed)
			Drug interactions not observed with agents metabolized by CYP3A4 or p-glycoprotein	

**Table 2:** In vitro spectrum of activity of antifungals currently under late-stage clinical development, including manogepix, olorofim, ibrexafungerp, rezafungin, and oteseconazole. + = in vitro antifungal activity observed; − = no in vitro activitiy; blank cells = unknown.

Antifungal	Manogepix	Olorofim	Ibrexafungerp	Rezafungin	Oteseconazole
Yeasts					
*C. albicans*	+	−	+	+	+
*C. auris*	+	−	+	+	+
*C. glabrata*	+	−	+	+	+
*C. krusei*	−	−	+	+	+
*C. parapsilosis*	+	−	+	+	+
*C. tropicalis*	+	−	+	+	+
*C. gattii*	+	−	−	−	+
*C. neoformans*	+	−	−	−	+
*Rhodotorula*	+	−	−	−	
*Trichosporon*	+/−	−	−	−	
*Aspergillus*					
*A. flavus*	+	+	+	+	−
*A. fumigatus*	+	+	+	+	−
*A. niger*	+	+	+	+	−
*A. terreus*	+	+	+	+	−
*Fusarium*					
*F. oxysporum*	+	+/−	−		
*F. solani*	+	−	−		
*Scedosporium*					
*Scedosporium*	+	+	−		
*L. prolificans*	+	+	−		
Mucorales					
*Mucor*	−	−	−	−	
*Rhizopus*	+/−	−	−	−	+/−
Other Mucorales	−	−	−	−	
Endemic Fungi					
*Blastomyces*		+	+		+
*Coccidioides*	+	+	+		+
*Histoplasma*		+	+		+
Dermatophytes					
*Trichophyton*		+			+

## Data Availability

Not applicable.

## References

[1] Lewis R.E. (2011). Current concepts in antifungal pharmacology. Mayo Clin. Proc..

[2] Ashley E.S.D., Lewis R., Lewis J.S., Martin C., Andes D. (2006). Pharmacology of systemic antifungal agents. Clin. Infect. Dis..

[3] Arastehfar A., Gabaldon T., Garcia-Rubio R., Jenks J.D., Hoenigl M., Salzer H.J.F., Ilkit M., Lass-Florl C., Perlin D.S. (2020). Drug-Resistant Fungi: An Emerging Challenge Threatening Our Limited Antifungal Armamentarium. Antibiotics.

[4] Hoenigl M., Sprute R., Arastehfar A., Perfect J.R., Lass-Florl C., Bellmann R., Prattes J., Thompson G.R., Wiederhold N.P., Al Obaidi M.M. (2022). Invasive candidiasis: Investigational drugs in the clinical development pipeline and mechanisms of action. Expert Opin. Investig. Drugs.

[5] Hoenigl M., Sprute R., Egger M., Arastehfar A., Cornely O.A., Krause R., Lass-Florl C., Prattes J., Spec A., Thompson G.R. (2021). The Antifungal Pipeline: Fosmanogepix, Ibrexafungerp, Olorofim, Opelconazole, and Rezafungin. Drugs.

[6] CLSI (2020). Performance Standards for Antifungal Susceptibility Testing of Filamentous Fungi.

[7] CLSI (2020). Performance Standards for Antifungal Susceptibility Testing of Yeasts.

[8] Miyazaki M., Horii T., Hata K., Watanabe N.A., Nakamoto K., Tanaka K., Shirotori S., Murai N., Inoue S., Matsukura M. (2011). In vitro activity of E1210, a novel antifungal, against clinically important yeasts and molds. Antimicrob. Agents Chemother..

[9] Tsukahara K., Hata K., Nakamoto K., Sagane K., Watanabe N.A., Kuromitsu J., Kai J., Tsuchiya M., Ohba F., Jigami Y. (2003). Medicinal genetics approach towards identifying the molecular target of a novel inhibitor of fungal cell wall assembly. Mol. Microbiol..

[10] Chaffin W.L. (2008). *Candida albicans* cell wall proteins. Microbiol. Mol. Biol. Rev. MMBR.

[11] Fu Y., Luo G., Spellberg B.J., Edwards J.E., Ibrahim A.S. (2008). Gene overexpression/suppression analysis of candidate virulence factors of *Candida albicans*. Eukaryot. Cell.

[12] Hoyer L.L. (2001). The ALS gene family of *Candida albicans*. Trends Microbiol..

[13] Kapteyn J.C., Hoyer L.L., Hecht J.E., Muller W.H., Andel A., Verkleij A.J., Makarow M., Van Den Ende H., Klis F.M. (2000). The cell wall architecture of *Candida albicans* wild-type cells and cell wall-defective mutants. Mol. Microbiol..

[14] Sheppard D.C., Yeaman M.R., Welch W.H., Phan Q.T., Fu Y., Ibrahim A.S., Filler S.G., Zhang M., Waring A.J., Edwards J.E. (2004). Functional and structural diversity in the Als protein family of *Candida albicans*. J. Biol. Chem..

[15] Shaw K.J., Ibrahim A.S. (2020). Fosmanogepix: A Review of the First-in-Class Broad Spectrum Agent for the Treatment of Invasive Fungal Infections. J. Fungi.

[16] Arendrup M.C., Jorgensen K.M. (2020). Manogepix (APX001A) Displays Potent In Vitro Activity against Human Pathogenic Yeast, but with an Unexpected Correlation to Fluconazole MICs. Antimicrob. Agents Chemother..

[17] Pfaller M.A., Huband M.D., Flamm R.K., Bien P.A., Castanheira M. (2021). Antimicrobial activity of manogepix, a first-in-class antifungal, and comparator agents tested against contemporary invasive fungal isolates from an international surveillance programme (2018–2019). J. Glob. Antimicrob. Resist..

[18] Arendrup M.C., Chowdhary A., Jorgensen K.M., Meletiadis J. (2020). Manogepix (APX001A) In Vitro Activity against *Candida auris*: Head-to-Head Comparison of EUCAST and CLSI MICs. Antimicrob. Agents Chemother..

[19] Pfaller M.A., Hata K., Jones R.N., Messer S.A., Moet G.J., Castanheira M. (2011). In vitro activity of a novel broad-spectrum antifungal, E1210, tested against *Candida* spp. as determined by CLSI broth microdilution method. Diagn. Microbiol. Infect. Dis..

[20] Wiederhold N.P., Najvar L.K., Shaw K.J., Jaramillo R., Patterson H., Olivo M., Catano G., Patterson T.F. (2019). Efficacy of Delayed Therapy with Fosmanogepix (APX001) in a Murine Model of *Candida auris* Invasive Candidiasis. Antimicrob. Agents Chemother..

[21] Shaw K.J., Schell W.A., Covel J., Duboc G., Giamberardino C., Kapoor M., Moloney M., Soltow Q.A., Tenor J.L., Toffaletti D.L. (2018). In Vitro and In Vivo Evaluation of APX001A/APX001 and Other Gwt1 Inhibitors against Cryptococcus. Antimicrob. Agents Chemother..

[22] Hager C.L., Larkin E.L., Long L., Zohra Abidi F., Shaw K.J., Ghannoum M.A. (2018). In Vitro and In Vivo Evaluation of the Antifungal Activity of APX001A/APX001 against *Candida auris*. Antimicrob. Agents Chemother..

[23] Zhao M., Lepak A.J., VanScoy B., Bader J.C., Marchillo K., Vanhecker J., Ambrose P.G., Andes D.R. (2018). In Vivo Pharmacokinetics and Pharmacodynamics of APX001 against *Candida* spp. in a Neutropenic Disseminated Candidiasis Mouse Model. Antimicrob. Agents Chemother..

[24] Alkhazraji S., Gebremariam T., Alqarihi A., Gu Y., Mamouei Z., Singh S., Wiederhold N.P., Shaw K.J., Ibrahim A.S. (2020). Fosmanogepix (APX001) Is Effective in the Treatment of Immunocompromised Mice Infected with Invasive Pulmonary Scedosporiosis or Disseminated Fusariosis. Antimicrob. Agents Chemother..

[25] Castanheira M., Duncanson F.P., Diekema D.J., Guarro J., Jones R.N., Pfaller M.A. (2012). Activities of E1210 and comparator agents tested by CLSI and EUCAST broth microdilution methods against Fusarium and Scedosporium species identified using molecular methods. Antimicrob. Agents Chemother..

[26] Gebremariam T., Alkhazraji S., Alqarihi A., Jeon H.H., Gu Y., Kapoor M., Shaw K.J., Ibrahim A.S. (2019). APX001 Is Effective in the Treatment of Murine Invasive Pulmonary Aspergillosis. Antimicrob. Agents Chemother..

[27] Hata K., Horii T., Miyazaki M., Watanabe N.A., Okubo M., Sonoda J., Nakamoto K., Tanaka K., Shirotori S., Murai N. (2011). Efficacy of oral E1210, a new broad-spectrum antifungal with a novel mechanism of action, in murine models of candidiasis, aspergillosis, and fusariosis. Antimicrob. Agents Chemother..

[28] Jorgensen K.M., Astvad K.M.T., Arendrup M.C. (2020). In Vitro Activity of Manogepix (APX001A) and Comparators against Contemporary Molds: MEC Comparison and Preliminary Experience with Colorimetric MIC Determination. Antimicrob. Agents Chemother..

[29] Pfaller M.A., Duncanson F., Messer S.A., Moet G.J., Jones R.N., Castanheira M. (2011). In vitro activity of a novel broad-spectrum antifungal, E1210, tested against *Aspergillus* spp. determined by CLSI and EUCAST broth microdilution methods. Antimicrob. Agents Chemother..

[30] Arikan S., Lozano-Chiu M., Paetznick V., Rex J.H. (2001). In vitro susceptibility testing methods for caspofungin against Aspergillus and Fusarium isolates. Antimicrob. Agents Chemother..

[31] Gebremariam T., Alkhazraji S., Alqarihi A., Wiederhold N.P., Shaw K.J., Patterson T.F., Filler S.G., Ibrahim A.S. (2020). Fosmanogepix (APX001) Is Effective in the Treatment of Pulmonary Murine Mucormycosis Due to *Rhizopus arrhizus*. Antimicrob. Agents Chemother..

[32] Gebremariam T., Gu Y., Alkhazraji S., Youssef E., Shaw K.J., Ibrahim A.S. (2022). The Combination Treatment of Fosmanogepix and Liposomal Amphotericin B Is Superior to Monotherapy in Treating Experimental Invasive Mold Infections. Antimicrob. Agents Chemother..

[33] Kapoor M., Moloney M., Soltow Q.A., Pillar C.M., Shaw K.J. (2019). Evaluation of Resistance Development to the Gwt1 Inhibitor Manogepix (APX001A) in Candida Species. Antimicrob. Agents Chemother..

[34] Liston S.D., Whitesell L., Kapoor M., Shaw K.J., Cowen L.E. (2020). Enhanced Efflux Pump Expression in Candida Mutants Results in Decreased Manogepix Susceptibility. Antimicrob. Agents Chemother..

[35] Zhao M., Lepak A.J., Marchillo K., Vanhecker J., Sanchez H., Ambrose P.G., Andes D.R. (2019). APX001 Pharmacokinetic/Pharmacodynamic Target Determination against *Aspergillus fumigatus* in an In Vivo Model of Invasive Pulmonary Aspergillosis. Antimicrob. Agents Chemother..

[36] Viriyakosol S., Kapoor M., Okamoto S., Covel J., Soltow Q.A., Trzoss M., Shaw K.J., Fierer J. (2019). APX001 and Other Gwt1 Inhibitor Prodrugs Are Effective in Experimental *Coccidioides immitis* Pneumonia. Antimicrob. Agents Chemother..

[37] Zhao Y., Lee M.H., Paderu P., Lee A., Jimenez-Ortigosa C., Park S., Mansbach R.S., Shaw K.J., Perlin D.S. (2018). Significantly Improved Pharmacokinetics Enhances In Vivo Efficacy of APX001 against Echinocandin- and Multidrug-Resistant Candida Isolates in a Mouse Model of Invasive Candidiasis. Antimicrob. Agents Chemother..

[38] Watanabe N.A., Miyazaki M., Horii T., Sagane K., Tsukahara K., Hata K. (2012). E1210, a new broad-spectrum antifungal, suppresses *Candida albicans* hyphal growth through inhibition of glycosylphosphatidylinositol biosynthesis. Antimicrob. Agents Chemother..

[39] Hodges M.R., Ople E., Shaw K.J., Mansbach R., Van Marle S.J., Van Hoogdalem E., Kramer W., Wedel P. (2017). Phase 1 Study to Assess Safety, Tolerability and Pharmacokinetics of Single and Multiple Oral Doses of APX001 and to Investigate the Effect of Food on APX001 Bioavailability. Open Forum Infect. Dis..

[40] Hodges M.R., Ople E., Shaw K.J., Mansbach R., Van Marle S.J., Van Hoogdalem E., Wedel P., Kramer W. (2017). First-in-Human Study to Assess Safety, Tolerability and Pharmacokinetics of APX001 Administered by Intravenous Infusion to Healthy Subjects. Open Forum Infect. Dis..

[41] Pappas P.G., Kullberg B.J., Vazquez J.A., Oren I., Rahav G., Aoun M., Bulpa P., Ben-Ami R., Ferrer R., McCarty T. Clinical Safety and Efficacy of Novel Antifungal, Fosmanogepix, in the Treatment of Candidemia: Results from a Phase 2 Proof of Concept Trial. Presented at the Programs and Abstracts of the 55th Annual Infectious Diseases Society of America (IDSA) Meeting.

[42] Bulpa P., Rahav G., Oren I., Aoun M., Thompson G.R., Pappas P.G., Kullberg B.J., Vazquez J.A., Barbat S.H., Wedel P. Clinical Safety and Efficacy of Fosmanogepix, a Novel First-in-class Antifungal, in Patients with Renal Insufficiency: Subset Analysis from a Phase 2 Candidemia Trial. Proceedings of the Programs and Abstracts of the 55th Annual Infectious Diseases Society of America (IDSA) Meeting.

[43] Balani S.K., Zhu T., Yang T.J., Liu Z., He B., Lee F.W. (2002). Effective dosing regimen of 1-aminobenzotriazole for inhibition of antipyrine clearance in rats, dogs, and monkeys. Drug Metab. Dispos..

[44] Oliver J.D., Sibley G.E., Beckmann N., Dobb K.S., Slater M.J., McEntee L., du Pre S., Livermore J., Bromley M.J., Wiederhold N.P. (2016). F901318 represents a novel class of antifungal drug that inhibits dihydroorotate dehydrogenase. Proc. Natl. Acad. Sci. USA.

[45] Gow N.A.R., Latge J.P., Munro C.A. (2017). The Fungal Cell Wall: Structure, Biosynthesis, and Function. Microbiol. Spectr..

[46] du Pre S., Beckmann N., Almeida M.C., Sibley G.E.M., Law D., Brand A.C., Birch M., Read N.D., Oliver J.D. (2018). Effect of the Novel Antifungal Drug F901318 (Olorofim) on Growth and Viability of *Aspergillus fumigatus*. Antimicrob. Agents Chemother..

[47] du Pre S., Birch M., Law D., Beckmann N., Sibley G.E.M., Bromley M.J., Read N.D., Oliver J.D. (2020). The Dynamic Influence of Olorofim (F901318) on the Cell Morphology and Organization of Living Cells of *Aspergillus fumigatus*. J. Fungi.

[48] Wiederhold N.P. (2020). Review of the Novel Investigational Antifungal Olorofim. J. Fungi.

[49] Wiederhold N.P., Law D., Birch M. (2017). Dihydroorotate dehydrogenase inhibitor F901318 has potent in vitro activity against Scedosporium species and Lomentospora prolificans. J. Antimicrob. Chemother..

[50] Wiederhold N.P., Najvar L.K., Jaramillo R., Olivo M., Birch M., Law D., Rex J.H., Catano G., Patterson T.F. (2018). The Orotomide Olorofim Is Efficacious in an Experimental Model of Central Nervous System Coccidioidomycosis. Antimicrob. Agents Chemother..

[51] Biswas C., Law D., Birch M., Halliday C., Sorrell T.C., Rex J., Slavin M., Chen S.C. (2018). In vitro activity of the novel antifungal compound F901318 against Australian Scedosporium and Lomentospora fungi. Med. Mycol..

[52] Jorgensen K.M., Astvad K.M.T., Hare R.K., Arendrup M.C. (2018). EUCAST Determination of Olorofim (F901318) Susceptibility of Mold Species, Method Validation, and MICs. Antimicrob. Agents Chemother..

[53] Rivero-Menendez O., Cuenca-Estrella M., Alastruey-Izquierdo A. (2019). In vitro activity of olorofim (F901318) against clinical isolates of cryptic species of Aspergillus by EUCAST and CLSI methodologies. J. Antimicrob. Chemother..

[54] Seyedmousavi S., Chang Y.C., Law D., Birch M., Rex J.H., Kwon-Chung K.J. (2019). Efficacy of Olorofim (F901318) against *Aspergillus fumigatus*, A. nidulans, and A. tanneri in Murine Models of Profound Neutropenia and Chronic Granulomatous Disease. Antimicrob. Agents Chemother..

[55] Buil J.B., Rijs A., Meis J.F., Birch M., Law D., Melchers W.J.G., Verweij P.E. (2017). In vitro activity of the novel antifungal compound F901318 against difficult-to-treat Aspergillus isolates. J. Antimicrob. Chemother..

[56] Lim W., Eadie K., Konings M., Rijnders B., Fahal A.H., Oliver J.D., Birch M., Verbon A., van de Sande W. (2020). Madurella mycetomatis, the main causative agent of eumycetoma, is highly susceptible to olorofim. J. Antimicrob. Chemother..

[57] Buil J.B., Oliver J.D., Law D., Baltussen T., Zoll J., Hokken M.W.J., Tehupeiory-Kooreman M., Melchers W.J.G., Birch M., Verweij P.E. (2022). Resistance profiling of *Aspergillus fumigatus* to olorofim indicates absence of intrinsic resistance and unveils the molecular mechanisms of acquired olorofim resistance. Emerg. Microbes Infect..

[58] Hope W.W., McEntee L., Livermore J., Whalley S., Johnson A., Farrington N., Kolamunnage-Dona R., Schwartz J., Kennedy A., Law D. (2017). Pharmacodynamics of the Orotomides against *Aspergillus fumigatus*: New Opportunities for Treatment of Multidrug-Resistant Fungal Disease. mBio.

[59] Negri C.E., Johnson A., McEntee L., Box H., Whalley S., Schwartz J.A., Ramos-Martin V., Livermore J., Kolamunnage-Dona R., Colombo A.L. (2018). Pharmacodynamics of the Novel Antifungal Agent F901318 for Acute Sinopulmonary Aspergillosis Caused by *Aspergillus flavus*. J. Infect. Dis..

[60] Seyedmousavi S., Chang Y.C., Youn J.H., Law D., Birch M., Rex J.H., Kwon-Chung K.J. (2021). In Vivo Efficacy of Olorofim against Systemic Scedosporiosis and Lomentosporiosis. Antimicrob. Agents Chemother..

[61] Kennedy T., Allen G., Steiner J., Heep M., Birch M. Assessment of the duration of infusion on the tolerability and repeat dose pharmacokinetics of F901318 in healthy volunteers (abstr. P1711). Proceedings of the 27th European Congress of Clinical Microbiology and Infectious Diseases.

[62] Kennedy T., Allen G., Steiner J., Heep M., Oliver J., Sibley G., Law D. Multiple dose pharmacokinetics of an immediate-release tablet formulation of F901318 in healthy male and female subjects (abstr. P1710). Proceedings of the 27th European Congress of Clinical Microbiology and Infectious Diseases.

[63] Rauseo A.M., Coler-Reilly A., Larson L., Spec A. (2020). Hope on the Horizon: Novel Fungal Treatments in Development. Open Forum Infect. Dis..

[64] Kennedy T., Allen G., Steiner J., Oliver J., Birch M., Sibley G., Law D. An open-label study in healthy volunteers to evaluate the potential for cytochrome P450 3A4 inhibition by F901318 using oral midazolam as a probe (abstr. P1737). Proceedings of the 27th European Congress of Clinical Microbiology and Infectious Diseases.

[65] Mitsuyama J., Nomura N., Hashimoto K., Yamada E., Nishikawa H., Kaeriyama M., Kimura A., Todo Y., Narita H. (2008). In vitro and in vivo antifungal activities of T-2307, a novel arylamidine. Antimicrob. Agents Chemother..

[66] Lanteri C.A., Trumpower B.L., Tidwell R.R., Meshnick S.R. (2004). DB75, a novel trypanocidal agent, disrupts mitochondrial function in Saccharomyces cerevisiae. Antimicrob. Agents Chemother..

[67] Yamada E., Nishikawa H., Nomura N., Mitsuyama J. (2010). T-2307 shows efficacy in a murine model of *Candida glabrata* infection despite in vitro trailing growth phenomena. Antimicrob. Agents Chemother..

[68] Yamashita K., Miyazaki T., Fukuda Y., Mitsuyama J., Saijo T., Shimamura S., Yamamoto K., Imamura Y., Izumikawa K., Yanagihara K. (2019). The Novel Arylamidine T-2307 Selectively Disrupts Yeast Mitochondrial Function by Inhibiting Respiratory Chain Complexes. Antimicrob. Agents Chemother..

[69] Wiederhold N.P., Najvar L.K., Jaramillo R., Olivo M., Patterson H., Connell A., Fukuda Y., Mitsuyama J., Catano G., Patterson T.F. (2020). The Novel Arylamidine T-2307 Demonstrates In Vitro and In Vivo Activity against *Candida auris*. Antimicrob. Agents Chemother..

[70] Wiederhold N.P., Najvar L.K., Fothergill A.W., Bocanegra R., Olivo M., McCarthy D.I., Fukuda Y., Mitsuyama J., Patterson T.F. (2016). The novel arylamidine T-2307 demonstrates in vitro and in vivo activity against echinocandin-resistant *Candida glabrata*. J. Antimicrob. Chemother..

[71] Wiederhold N.P., Najvar L.K., Fothergill A.W., Bocanegra R., Olivo M., McCarthy D.I., Kirkpatrick W.R., Fukuda Y., Mitsuyama J., Patterson T.F. (2015). The novel arylamidine T-2307 maintains in vitro and in vivo activity against echinocandin-resistant *Candida albicans*. Antimicrob. Agents Chemother..

[72] Nishikawa H., Fukuda Y., Mitsuyama J., Tashiro M., Tanaka A., Takazono T., Saijo T., Yamamoto K., Nakamura S., Imamura Y. (2017). In vitro and in vivo antifungal activities of T-2307, a novel arylamidine, against *Cryptococcus gattii*: An emerging fungal pathogen. J. Antimicrob. Chemother..

[73] Abe M., Nakamura S., Kinjo Y., Masuyama Y., Mitsuyama J., Kaku M., Miyazaki Y. (2019). Efficacy of T-2307, a novel arylamidine, against ocular complications of disseminated candidiasis in mice. J. Antimicrob. Chemother..

[74] Wiederhold N.P., Najvar L.K., Bocanegra R., Kirkpatrick W.R., Patterson T.F. (2012). Comparison of anidulafungin’s and fluconazole’s in vivo activity in neutropenic and non-neutropenic models of invasive candidiasis. Clin. Microbiol. Infect..

[75] Nakamura I., Yoshimura S., Masaki T., Takase S., Ohsumi K., Hashimoto M., Furukawa S., Fujie A. (2017). ASP2397: A novel antifungal agent produced by *Acremonium persicinum* MF-347833. J. Antibiot..

[76] Nakamura I., Ohsumi K., Takeda S., Katsumata K., Matsumoto S., Akamatsu S., Mitori H., Nakai T. (2019). ASP2397 Is a Novel Natural Compound That Exhibits Rapid and Potent Fungicidal Activity against Aspergillus Species through a Specific Transporter. Antimicrob. Agents Chemother..

[77] Dietl A.M., Misslinger M., Aguiar M.M., Ivashov V., Teis D., Pfister J., Decristoforo C., Hermann M., Sullivan S.M., Smith L.R. (2019). The Siderophore Transporter Sit1 Determines Susceptibility to the Antifungal VL-2397. Antimicrob. Agents Chemother..

[78] Wiederhold N.P. (2018). The antifungal arsenal: Alternative drugs and future targets. Int. J. Antimicrob. Agents.

[79] Arendrup M.C., Jensen R.H., Cuenca-Estrella M. (2016). In Vitro Activity of ASP2397 against Aspergillus Isolates with or without Acquired Azole Resistance Mechanisms. Antimicrob. Agents Chemother..

[80] Rubino C.M., Smith L.R., Mammen M.P., Hopkins A.M., Lakota E.A., Sullivan S.M. (2017). Pharmacokinetic-Pharmacodynamic Target Attainment Analysis to Support VL-2397 Dose Selection for a Phase 2 Trial in Patients with Invasive Aspergillosis. Open Forum Infect. Dis..

[81] Pfaller M.A., Messer S.A., Motyl M.R., Jones R.N., Castanheira M. (2013). Activity of MK-3118, a new oral glucan synthase inhibitor, tested against *Candida* spp. by two international methods (CLSI and EUCAST). J. Antimicrob. Chemother..

[82] Bowman J.C., Hicks P.S., Kurtz M.B., Rosen H., Schmatz D.M., Liberator P.A., Douglas C.M. (2002). The antifungal echinocandin caspofungin acetate kills growing cells of *Aspergillus fumigatus* in vitro. Antimicrob. Agents Chemother..

[83] Ghannoum M., Long L., Larkin E.L., Isham N., Sherif R., Borroto-Esoda K., Barat S., Angulo D. (2018). Evaluation of the Antifungal Activity of the Novel Oral Glucan Synthase Inhibitor SCY-078, Singly and in Combination, for the Treatment of Invasive Aspergillosis. Antimicrob. Agents Chemother..

[84] Douglas C.M., D’Ippolito J.A., Shei G.J., Meinz M., Onishi J., Marrinan J.A., Li W., Abruzzo G.K., Flattery A., Bartizal K. (1997). Identification of the *FKS1* gene of *Candida albicans* as the essential target of 1,3-beta-D-glucan synthase inhibitors. Antimicrob. Agents Chemother..

[85] Pfaller M.A., Messer S.A., Motyl M.R., Jones R.N., Castanheira M. (2013). In vitro activity of a new oral glucan synthase inhibitor (MK-3118) tested against *Aspergillus* spp. by CLSI and EUCAST broth microdilution methods. Antimicrob. Agents Chemother..

[86] Walker S.S., Xu Y., Triantafyllou I., Waldman M.F., Mendrick C., Brown N., Mann P., Chau A., Patel R., Bauman N. (2011). Discovery of a novel class of orally active antifungal beta-1,3-D-glucan synthase inhibitors. Antimicrob. Agents Chemother..

[87] Jiménez-Ortigosa C., Perez W.B., Angulo D., Borroto-Esoda K., Perlin D.S. (2017). De Novo Acquisition of Resistance to SCY-078 in *Candida glabrata* Involves FKS Mutations That both Overlap and Are Distinct from Those Conferring Echinocandin Resistance. Antimicrob. Agents Chemother..

[88] Schell W.A., Jones A.M., Borroto-Esoda K., Alexander B.D. (2017). Antifungal Activity of SCY-078 and Standard Antifungal Agents against 178 Clinical Isolates of Resistant and Susceptible Candida Species. Antimicrob. Agents Chemother..

[89] Larkin E., Hager C., Chandra J., Mukherjee P.K., Retuerto M., Salem I., Long L., Isham N., Kovanda L., Borroto-Esoda K. (2017). The Emerging Pathogen *Candida auris*: Growth Phenotype, Virulence Factors, Activity of Antifungals, and Effect of SCY-078, a Novel Glucan Synthesis Inhibitor, on Growth Morphology and Biofilm Formation. Antimicrob. Agents Chemother..

[90] Berkow E.L., Angulo D., Lockhart S.R. (2017). In Vitro activity of a novel glucan synthase inhibitor, SCY-078, against clinical isolates of *Candida auris*. Antimicrob. Agents Chemother..

[91] Larkin E.L., Long L., Isham N., Borroto-Esoda K., Barat S., Angulo D., Wring S., Ghannoum M. (2019). A Novel 1,3-Beta-d-Glucan Inhibitor, Ibrexafungerp (Formerly SCY-078), Shows Potent Activity in the Lower pH Environment of Vulvovaginitis. Antimicrob. Agents Chemother..

[92] Danby C.S., Boikov D., Rautemaa-Richardson R., Sobel J.D. (2012). Effect of pH on in vitro susceptibility of *Candida glabrata* and *Candida albicans* to 11 antifungal agents and implications for clinical use. Antimicrob. Agents Chemother..

[93] Spitzer M., Wiederhold N.P. (2018). Reduced Antifungal Susceptibility of Vulvovaginal Candida Species at Normal Vaginal pH Levels: Clinical Implications. J. Low. Genit. Tract Dis..

[94] Rivero-Menendez O., Soto-Debran J.C., Cuenca-Estrella M., Alastruey-Izquierdo A. (2021). In Vitro Activity of Ibrexafungerp against a Collection of Clinical Isolates of Aspergillus, Including Cryptic Species and Cyp51A Mutants, Using EUCAST and CLSI Methodologies. J. Fungi.

[95] Jiménez-Ortigosa C., Paderu P., Motyl M.R., Perlin D.S. (2014). Enfumafungin derivative MK-3118 shows increased in vitro potency against clinical echinocandin-resistant Candida Species and Aspergillus species isolates. Antimicrob. Agents Chemother..

[96] Petraitis V., Petraitiene R., Katragkou A., Maung B.B.W., Naing E., Kavaliauskas P., Barat S., Borroto-Esoda K., Azie N., Angulo D. (2020). Combination Therapy with Ibrexafungerp (Formerly SCY-078), a First-in-Class Triterpenoid Inhibitor of (1—>3)-beta-d-Glucan Synthesis, and Isavuconazole for Treatment of Experimental Invasive Pulmonary Aspergillosis. Antimicrob. Agents Chemother..

[97] Lamoth F., Alexander B.D. (2015). Antifungal activities of SCY-078 (MK-3118) and standard antifungal agents against clinical non-Aspergillus mold isolates. Antimicrob. Agents Chemother..

[98] Park S., Kelly R., Kahn J.N., Robles J., Hsu M.J., Register E., Li W., Vyas V., Fan H., Abruzzo G. (2005). Specific substitutions in the echinocandin target Fks1p account for reduced susceptibility of rare laboratory and clinical *Candida* sp. isolates. Antimicrob. Agents Chemother..

[99] Nunnally N.S., Etienne K.A., Angulo D., Lockhart S.R., Berkow E.L. (2019). In Vitro Activity of Ibrexafungerp, a Novel Glucan Synthase Inhibitor against *Candida glabrata* Isolates with FKS Mutations. Antimicrob. Agents Chemother..

[100] Jallow S., Govender N.P. (2021). Ibrexafungerp: A First-in-Class Oral Triterpenoid Glucan Synthase Inhibitor. J. Fungi.

[101] Wiederhold N.P., Najvar L.K., Jaramillo R., Olivo M., Pizzini J., Catano G., Patterson T.F. (2018). Oral glucan synthase inhibitor SCY-078 is effective in an experimental murine model of invasive candidiasis caused by WT and echinocandin-resistant *Candida glabrata*. J. Antimicrob. Chemother..

[102] Zhu Y.C., Barat S.A., Borroto-Esoda K., Angulo D., Chaturvedi S., Chaturvedi V. (2020). Pan-resistant *Candida auris* isolates from the outbreak in New York are susceptible to ibrexafungerp (a glucan synthase inhibitor). Int. J. Antimicrob. Agents.

[103] Ghannoum M., Long L., Isham N., Hager C., Wilson R., Borroto-Esoda K., Barat S., Angulo D. (2019). Activity of a novel 1,3-beta-D-glucan Synthase Inhibitor, Ibrexafungerp (formerly SCY-078), Against *Candida glabrata*. Antimicrob. Agents Chemother..

[104] Marcos-Zambrano L.J., Gomez-Perosanz M., Escribano P., Bouza E., Guinea J. (2017). The novel oral glucan synthase inhibitor SCY-078 shows in vitro activity against sessile and planktonic *Candida* spp.. J. Antimicrob. Chemother..

[105] Pfaller M.A., Messer S.A., Rhomberg P.R., Borroto-Esoda K., Castanheira M. (2017). Differential Activity of the Oral Glucan Synthase Inhibitor SCY-078 against Wild-Type and Echinocandin-Resistant Strains of Candida Species. Antimicrob. Agents Chemother..

[106] Mesquida A., Diaz-Garcia J., Sanchez-Carrillo C., Martin-Rabadan P., Alcala L., Munoz P., Escribano P., Guinea J. (2022). ΔF659 and F659S substitutions at the HS1 of *FKS2* gene, along with E655A and W715L upstream and downstream substitutions, correlate with high ibrexafungerp MICs against *Candida glabrata*. Clin. Microbiol. Infect..

[107] Lepak A.J., Marchillo K., Andes D.R. (2015). Pharmacodynamic target evaluation of a novel oral glucan synthase inhibitor, SCY-078 (MK-3118), using an in vivo murine invasive candidiasis model. Antimicrob. Agents Chemother..

[108] Wiederhold N.P., Najvar L.K., Olivo M., Morris K.N., Patterson H.P., Catano G., Patterson T.F. (2021). Ibrexafungerp Demonstrates In Vitro Activity against Fluconazole-Resistant *Candida auris* and In Vivo Efficacy with Delayed Initiation of Therapy in an Experimental Model of Invasive Candidiasis. Antimicrob. Agents Chemother..

[109] Barat S.A., Borroto-Esoda K., Angulo D., Ashbaugh A., Cushion M. Efficacy of Ibrexafungerp (formerly SCY-078) in a Murine Treatment Model of Pneumocystis Pneumonia. Proceedings of the ASM Microbe.

[110] Wring S.A., Randolph R., Park S., Abruzzo G., Chen Q., Flattery A., Garrett G., Peel M., Outcalt R., Powell K. (2017). Preclinical Pharmacokinetics and Pharmacodynamic Target of SCY-078, a First-in-Class Orally Active Antifungal Glucan Synthesis Inhibitor, in Murine Models of Disseminated Candidiasis. Antimicrob. Agents Chemother..

[111] Andes D., Diekema D.J., Pfaller M.A., Bohrmuller J., Marchillo K., Lepak A. (2010). In vivo comparison of the pharmacodynamic targets for echinocandin drugs against Candida species. Antimicrob. Agents Chemother..

[112] Davis M.R., Donnelley M.A., Thompson G.R. (2020). Ibrexafungerp: A novel oral glucan synthase inhibitor. Med. Mycol..

[113] Trucksis M., Garrett G., Heriman I. A phase I multiple rising dose study evaluating safety, tolerability, and pharmacokinetics of MK-3118, oral glucan synthase inhibitor, in healthy volunteers. Proceedings of the 51st Interscience Conference on Antimicrobial Agents and Chemotherapy (ICAAC).

[114] Alexander B.D., Cornely O., Pappas P., Miller R., Vazquez J.A., Ostrosky-Zeichner L., Spec A., Rautemaa-Richardson R., Krause R., Thompson III G.R. (2020). 1248. Efficacy and Safety of Oral Ibrexafungerp in 41 Patients with Refractory Fungal Diseases, Interim Analysis of a Phase 3 Open-label Study (FURI). Open Forum Infect. Dis..

[115] Spec A., Pullman J., Thompson G.R., Powderly W.G., Tobin E.H., Vazquez J., Wring S.A., Angulo D., Helou S., Pappas P.G. (2019). MSG-10: A Phase 2 study of oral ibrexafungerp (SCY-078) following initial echinocandin therapy in non-neutropenic patients with invasive candidiasis. J. Antimicrob. Chemother..

[116] Wring S., Murphy G., Atiee G., Corr C., Hyman M., Willett M., Angulo D. (2018). Lack of Impact by SCY-078, a First-in-Class Oral Fungicidal Glucan Synthase Inhibitor, on the Pharmacokinetics of Rosiglitazone, a Substrate for CYP450 2C8, Supports the Low Risk for Clinically Relevant Metabolic Drug-Drug Interactions. J. Clin. Pharmacol..

[117] Garcia-Effron G. (2020). Rezafungin-Mechanisms of Action, Susceptibility and Resistance: Similarities and Differences with the Other Echinocandins. J. Fungi.

[118] James K.D., Laudeman C.P., Malkar N.B., Krishnan R., Polowy K. (2017). Structure-Activity Relationships of a Series of Echinocandins and the Discovery of CD101, a Highly Stable and Soluble Echinocandin with Distinctive Pharmacokinetic Properties. Antimicrob. Agents Chemother..

[119] Krishnan B.R., James K.D., Polowy K., Bryant B.J., Vaidya A., Smith S., Laudeman C.P. (2017). CD101, a novel echinocandin with exceptional stability properties and enhanced aqueous solubility. J. Antibiot..

[120] Ong V., James K.D., Smith S., Krishnan B.R. (2017). Pharmacokinetics of the Novel Echinocandin CD101 in Multiple Animal Species. Antimicrob. Agents Chemother..

[121] Sandison T., Ong V., Lee J., Thye D. (2017). Safety and Pharmacokinetics of CD101 IV, a Novel Echinocandin, in Healthy Adults. Antimicrob. Agents Chemother..

[122] Krause D.S., Reinhardt J., Vazquez J.A., Reboli A., Goldstein B.P., Wible M., Henkel T., Anidulafungin Invasive Candidiasis Study Group (2004). Phase 2, randomized, dose-ranging study evaluating the safety and efficacy of anidulafungin in invasive candidiasis and candidemia. Antimicrob. Agents Chemother..

[123] Pfaller M.A., Messer S.A., Rhomberg P.R., Jones R.N., Castanheira M. (2016). Activity of a long-acting echinocandin, CD101, determined using CLSI and EUCAST reference methods, against *Candida* and *Aspergillus* spp., including echinocandin- and azole-resistant isolates. J. Antimicrob. Chemother..

[124] Pfaller M.A., Messer S.A., Rhomberg P.R., Castanheira M. (2017). CD101, a long-acting echinocandin, and comparator antifungal agents tested against a global collection of invasive fungal isolates in the SENTRY 2015 Antifungal Surveillance Program. Int. J. Antimicrob. Agents.

[125] Arendrup M.C., Meletiadis J., Zaragoza O., Jørgensen K.M., Marcos-Zambrano L.J., Kanioura L., Cuenca-Estrella M., Mouton J.W., Guinea J. (2018). Multicentre determination of rezafungin (CD101) susceptibility of Candida species by the EUCAST method. Clin. Microbiol. Infect..

[126] Tóth Z., Forgács L., Locke J.B., Kardos G., Nagy F., Kovács R., Szekely A., Borman A.M., Majoros L. (2019). In vitro activity of rezafungin against common and rare Candida species and Saccharomyces cerevisiae. J. Antimicrob. Chemother..

[127] Wiederhold N.P., Locke J.B., Daruwala P., Bartizal K. (2018). Rezafungin (CD101) demonstrates potent in vitro activity against Aspergillus, including azole-resistant *Aspergillus fumigatus* isolates and cryptic species. J. Antimicrob. Chemother..

[128] Dudiuk C., Macedo D., Leonardelli F., Theill L., Cabeza M.S., Gamarra S., Garcia-Effron G. (2017). Molecular Confirmation of the Relationship between *Candida guilliermondii* Fks1p Naturally Occurring Amino Acid Substitutions and Its Intrinsic Reduced Echinocandin Susceptibility. Antimicrob. Agents Chemother..

[129] Garcia-Effron G., Katiyar S.K., Park S., Edlind T.D., Perlin D.S. (2008). A naturally occurring proline-to-alanine amino acid change in Fks1p in *Candida parapsilosis*, *Candida orthopsilosis*, and *Candida metapsilosis* accounts for reduced echinocandin susceptibility. Antimicrob. Agents Chemother..

[130] Helleberg M., Jørgensen K.M., Hare R.K., Datcu R., Chowdhary A., Arendrup M.C. (2020). Rezafungin In Vitro Activity against Contemporary Nordic Clinical Candida Isolates and *Candida auris* Determined by the EUCAST Reference Method. Antimicrob. Agents Chemother..

[131] Lepak A.J., Zhao M., Andes D.R. (2018). Pharmacodynamic Evaluation of Rezafungin (CD101) against *Candida auris* in the Neutropenic Mouse Invasive Candidiasis Model. Antimicrob. Agents Chemother..

[132] Lepak A.J., Zhao M., Andes D.R. (2019). Determination of Pharmacodynamic Target Exposures for Rezafungin against *Candida tropicalis* and *Candida dubliniensis* in the Neutropenic Mouse Disseminated Candidiasis Model. Antimicrob. Agents Chemother..

[133] Hager C.L., Larkin E.L., Long L.A., Ghannoum M.A. (2018). Evaluation of the efficacy of rezafungin, a novel echinocandin, in the treatment of disseminated *Candida auris* infection using an immunocompromised mouse model. J. Antimicrob. Chemother..

[134] Zhao Y., Prideaux B., Nagasaki Y., Lee M.H., Chen P.Y., Blanc L., Ho H., Clancy C.J., Nguyen M.H., Dartois V. (2017). Unraveling Drug Penetration of Echinocandin Antifungals at the Site of Infection in an Intra-abdominal Abscess Model. Antimicrob. Agents Chemother..

[135] Zhao Y., Perez W.B., Jimenez-Ortigosa C., Hough G., Locke J.B., Ong V., Bartizal K., Perlin D.S. (2016). CD101: A novel long-acting echinocandin. Cell. Microbiol..

[136] Long L., Herrada J., Caley D., Munguba G., Sherif R., Bartizal K., Ghannoum M.A. Evaluation of the efficacy of rezafungin in the treatment of *Candida albicans* endophthalmitis using a rabbit model. Proceedings of the European Congress of Clinical Microbilogy and Infectious Diseases.

[137] Miesel L., Lin K.Y., Ong V. (2019). Rezafungin treatment in mouse models of invasive candidiasis and aspergillosis: Insights on the PK/PD pharmacometrics of rezafungin efficacy. Pharmacol. Res. Perspect..

[138] Wiederhold N.P., Najvar L.K., Jaramillo R., Olivo M., Wickes B.L., Catano G., Patterson T.F. (2019). Extended-Interval Dosing of Rezafungin against Azole-Resistant *Aspergillus fumigatus*. Antimicrob. Agents Chemother..

[139] Cushion M.T., Ashbaugh A. (2021). The Long-Acting Echinocandin, Rezafungin, Prevents Pneumocystis Pneumonia and Eliminates Pneumocystis from the Lungs in Prophylaxis and Murine Treatment Models. J. Fungi.

[140] Lakota E.A., Bader J.C., Ong V., Bartizal K., Miesel L., Andes D.R., Bhavnani S.M., Rubino C.M., Ambrose P.G., Lepak A.J. (2017). Pharmacological Basis of CD101 Efficacy: Exposure Shape Matters. Antimicrob. Agents Chemother..

[141] Wiederhold N.P., Kontoyiannis D.P., Chi J., Prince R.A., Tam V.H., Lewis R.E. (2004). Pharmacodynamics of caspofungin in a murine model of invasive pulmonary aspergillosis: Evidence of concentration-dependent activity. J. Infect. Dis..

[142] Flanagan S., Goodman D.B., Jandourek A., O’Reilly T., Sandison T. (2020). Lack of Effect of Rezafungin on QT/QTc Interval in Healthy Subjects. Clin. Pharmacol. Drug Dev..

[143] Thompson G.R., Soriano A., Skoutelis A., Vazquez J.A., Honore P.M., Horcajada J.P., Spapen H., Bassetti M., Ostrosky-Zeichner L., Das A.F. (2021). Rezafungin versus Caspofungin in a Phase 2, Randomized, Double-Blind Study for the Treatment of Candidemia and Invasive Candidiasis-The STRIVE Trial. Clin. Infect. Dis..

[144] Ong V., Hough G., Schlosser M., Bartizal K., Balkovec J.M., James K.D., Krishnan B.R. (2016). Preclinical Evaluation of the Stability, Safety, and Efficacy of CD101, a Novel Echinocandin. Antimicrob. Agents Chemother..

[145] Hoekstra W.J., Garvey E.P., Moore W.R., Rafferty S.W., Yates C.M., Schotzinger R.J. (2014). Design and optimization of highly-selective fungal CYP51 inhibitors. Bioorg. Med. Chem. Lett..

[146] Warrilow A.G., Hull C.M., Parker J.E., Garvey E.P., Hoekstra W.J., Moore W.R., Schotzinger R.J., Kelly D.E., Kelly S.L. (2014). The clinical candidate VT-1161 is a highly potent inhibitor of Candida albicans CYP51 but fails to bind the human enzyme. Antimicrob. Agents Chemother..

[147] Warrilow A.G., Parker J.E., Price C.L., Nes W.D., Garvey E.P., Hoekstra W.J., Schotzinger R.J., Kelly D.E., Kelly S.L. (2016). The Investigational Drug VT-1129 Is a Highly Potent Inhibitor of Cryptococcus Species CYP51 but Only Weakly Inhibits the Human Enzyme. Antimicrob. Agents Chemother..

[148] Warrilow A.G.S., Parker J.E., Price C.L., Garvey E.P., Hoekstra W.J., Schotzinger R.J., Wiederhold N.P., Nes W.D., Kelly D.E., Kelly S.L. (2017). The Tetrazole VT-1161 Is a Potent Inhibitor of Trichophyton rubrum through Its Inhibition of T. rubrum CYP51. Antimicrob. Agents Chemother..

[149] Shubitz L.F., Trinh H.T., Galgiani J.N., Lewis M.L., Fothergill A.W., Wiederhold N.P., Barker B.M., Lewis E.R., Doyle A.L., Hoekstra W.J. (2015). Evaluation of VT-1161 for Treatment of Coccidioidomycosis in Murine Infection Models. Antimicrob. Agents Chemother..

[150] Lockhart S.R., Fothergill A.W., Iqbal N., Bolden C.B., Grossman N.T., Garvey E.P., Brand S.R., Hoekstra W.J., Schotzinger R.J., Ottinger E. (2016). The Investigational Fungal Cyp51 Inhibitor VT-1129 Demonstrates Potent In Vitro Activity against *Cryptococcus neoformans* and *Cryptococcus gattii*. Antimicrob. Agents Chemother..

[151] Nishimoto A.T., Whaley S.G., Wiederhold N.P., Zhang Q., Yates C.M., Hoekstra W.J., Schotzinger R.J., Garvey E.P., Rogers P.D. (2019). Impact of the Major *Candida glabrata* Triazole Resistance Determinants on the Activity of the Novel Investigational Tetrazoles VT-1598 and VT-1161. Antimicrob. Agents Chemother..

[152] Nishimoto A.T., Wiederhold N.P., Flowers S.A., Zhang Q., Kelly S.L., Morschhauser J., Yates C.M., Hoekstra W.J., Schotzinger R.J., Garvey E.P. (2019). In Vitro Activities of the Novel Investigational Tetrazoles VT-1161 and VT-1598 Compared to the Triazole Antifungals against Azole-Resistant Strains and Clinical Isolates of *Candida albicans*. Antimicrob. Agents Chemother..

[153] Schell W.A., Jones A.M., Garvey E.P., Hoekstra W.J., Schotzinger R.J., Alexander B.D. (2017). Fungal CYP51 Inhibitors VT-1161 and VT-1129 Exhibit Strong In Vitro Activity against *Candida glabrata* and *C. krusei* Isolates Clinically Resistant to Azole and Echinocandin Antifungal Compounds. Antimicrob. Agents Chemother..

[154] Monk B.C., Keniya M.V., Sabherwal M., Wilson R.K., Graham D.O., Hassan H.F., Chen D., Tyndall J.D.A. (2019). Azole Resistance Reduces Susceptibility to the Tetrazole Antifungal VT-1161. Antimicrob. Agents Chemother..

[155] Garvey E.P., Hoekstra W.J., Schotzinger R.J., Sobel J.D., Lilly E.A., Fidel P.L. (2015). Efficacy of the clinical agent VT-1161 against fluconazole-sensitive and -resistant *Candida albicans* in a murine model of vaginal candidiasis. Antimicrob. Agents Chemother..

[156] Garvey E.P., Hoekstra W.J., Moore W.R., Schotzinger R.J., Long L., Ghannoum M.A. (2015). VT-1161 dosed once daily or once weekly exhibits potent efficacy in treatment of dermatophytosis in a guinea pig model. Antimicrob. Agents Chemother..

[157] Break T.J., Desai J.V., Natarajan M., Ferre E.M.N., Henderson C., Zelazny A.M., Siebenlist U., Hoekstra W.J., Schotzinger R.J., Garvey E.P. (2018). VT-1161 protects mice against oropharyngeal candidiasis caused by fluconazole-susceptible and -resistant *Candida albicans*. J. Antimicrob. Chemother..

[158] Wiederhold N.P., Shubitz L.F., Najvar L.K., Jaramillo R., Olivo M., Catano G., Trinh H.T., Yates C.M., Schotzinger R.J., Garvey E.P. (2018). The Novel Fungal Cyp51 Inhibitor VT-1598 Is Efficacious in Experimental Models of Central Nervous System Coccidioidomycosis Caused by *Coccidioides posadasii* and *Coccidioides immitis*. Antimicrob. Agents Chemother..

[159] Gebremariam T., Wiederhold N.P., Fothergill A.W., Garvey E.P., Hoekstra W.J., Schotzinger R.J., Patterson T.F., Filler S.G., Ibrahim A.S. (2015). VT-1161 Protects Immunosuppressed Mice from *Rhizopus arrhizus* var. arrhizus Infection. Antimicrob. Agents Chemother..

[160] Vivjoa (2022). Vivjoa (Oteseconazole) Package Insert.

[161] Sobel J.D., Nyirjesy P. (2021). Oteseconazole: An advance in treatment of recurrent vulvovaginal candidiasis. Future Microbiol..

[162] Brand S.R., Degenhardt T.P., Person K., Sobel J.D., Nyirjesy P., Schotzinger R.J., Tavakkol A. (2018). A phase 2, randomized, double-blind, placebo-controlled, dose-ranging study to evaluate the efficacy and safety of orally administered VT-1161 in the treatment of recurrent vulvovaginal candidiasis. Am. J. Obstet. Gynecol..

[163] Brand S.R., Sobel J.D., Nyirjesy P., Ghannoum M.A., Schotzinger R.J., Degenhardt T.P. (2021). A Randomized Phase 2 Study of VT-1161 for the Treatment of Acute Vulvovaginal Candidiasis. Clin. Infect. Dis..

[164] Elewski B., Brand S., Degenhardt T., Curelop S., Pollak R., Schotzinger R., Tavakkol A. (2021). A phase II, randomized, double-blind, placebo-controlled, dose-ranging study to evaluate the efficacy and safety of VT-1161 oral tablets in the treatment of patients with distal and lateral subungual onychomycosis of the toenail. Br. J. Dermatol..

[165] Cass L., Murray A., Davis A., Woodward K., Albayaty M., Ito K., Strong P., Ayrton J., Brindley C., Prosser J. (2021). Safety and nonclinical and clinical pharmacokinetics of PC945, a novel inhaled triazole antifungal agent. Pharmacol. Res. Perspect..

[166] Colley T., Alanio A., Kelly S.L., Sehra G., Kizawa Y., Warrilow A.G.S., Parker J.E., Kelly D.E., Kimura G., Anderson-Dring L. (2017). In Vitro and In Vivo Antifungal Profile of a Novel and Long-Acting Inhaled Azole, PC945, on *Aspergillus fumigatus* Infection. Antimicrob. Agents Chemother..

[167] Santangelo R., Paderu P., Delmas G., Chen Z.W., Mannino R., Zarif L., Perlin D.S. (2000). Efficacy of oral cochleate-amphotericin B in a mouse model of systemic candidiasis. Antimicrob. Agents Chemother..

[168] Kibathi L., Kumar P., Lionakis M., Urban A., Ferre E., McManus M., Colton B., Lambros C., Lu R., Mannino R. (2018). A phase lla efficacy safety, tolerability and pharmacokinetic study of encochleated amphotericin B in patients with mucocutaneous (esophageal, oropharyngeal, vulvovaginal) Candidiasis who are refractory or intolerant to standard non-intravenous therapies. Open Forum Infect. Dis..

